# 182 The Impact of Physical Activity on Obesity and NCD Outcomes: Insights from Kenyan Panel Data

**DOI:** 10.1093/eurpub/ckae114.115

**Published:** 2024-09-26

**Authors:** Cecilia Mainaa

**Affiliations:** Center for Development Research ZEF-University of Bonn, Bonn, Germany

## Abstract

Physical activity (PA) levels and sedentary behaviors are recognized as important modifiable risk factors for overweight, obesity, and non-communicable diseases (NCDs). However, there is a scarcity of evidence regarding the effects of different types of physical activities and sedentary time on these health outcomes in Sub-Saharan Africa. Employing a three-stage cluster sample design to gather panel data from 166 adults in Kenya, this study aims to investigate the empirical association between work-related PA, leisure PA, transport-related PA, and sedentary time with weight and the likelihood of an NCD outcome (either high blood pressure, diabetes, heart attack, or stroke). The main research questions are 1) what is the impact of work, leisure and transport-related PA on BMI? 2) to what extent does an increase in sedentary time contribute to a higher BMI? 3) what is the impact of work, leisure and transport-related physical activities on NCD outcomes? And finally, 4) to what extent does increased sedentary time contribute to a higher probability of an NCD outcome? The use of panel data from 2015 and 2022, allows us to account for time-invariant unobserved heterogeneity; additionally, findings were supported through robustness checks using entropy balancing.

Our results confirm a declining trend in PA levels and an increase in daily sedentary time, coupled with an overall increase in average BMI and incidences of NCDs. Using panel fixed effects, we find an increase of one metabolic equivalent (MET) hour per week in vigorous work activity is linked to a 0.025% reduction in BMI. Similarly, a comparable increase in leisure-related physical activity is associated with a BMI decrease of 0.16%, while an equivalent increase in transport-related activity results in a BMI reduction of 0.052%. We also find that work-related PA leads to a decrease in the probability of an NCD outcome, while sedentary time is positively associated with the probability of developing an NCD. Policies need to aim at promoting physical activity in the workplace and providing infrastructure that supports leisure and transport-related physical activities. Addressing these factors can play a crucial role in reducing the prevalence of obesity and NCDs among adults in Kenya.

